# Immediate Adverse Reactions to Gadolinium-Based MR Contrast Media: A Retrospective Analysis on 10,608 Examinations

**DOI:** 10.1155/2016/3918292

**Published:** 2016-08-29

**Authors:** Vincenza Granata, Marco Cascella, Roberta Fusco, Nicoletta dell'Aprovitola, Orlando Catalano, Salvatore Filice, Vincenzo Schiavone, Francesco Izzo, Arturo Cuomo, Antonella Petrillo

**Affiliations:** ^1^Department of Diagnostic Imaging, Radiant and Metabolic Therapy, Istituto Nazionale Tumori “Fondazione G. Pascale” IRCCS, 80131 Naples, Italy; ^2^Department of Anesthesia, Endoscopy and Cardiology, Istituto Nazionale Tumori “Fondazione G. Pascale” IRCCS, 80131 Naples, Italy; ^3^UOC Anestesia e Terapia Intensiva, Presidio Ospedaliero “Pineta Grande” Castel Volturno, 81100 Caserta, Italy; ^4^Department of Hepatobiliary Surgical Oncology, Istituto Nazionale Tumori “Fondazione G. Pascale” IRCCS, 80131 Naples, Italy

## Abstract

*Background and Purpose*. Contrast media (CM) for magnetic resonance imaging (MRI) may determine the development of acute adverse reactions. Objective was to retrospectively assess the frequency and severity of adverse reactions associated with gadolinium-based contrast agents (GBCAs) injection in patients who underwent MRI.* Material and Methods*. At our center 10608 MRI examinations with CM were performed using five different GBCAs: Gd-BOPTA (MultiHance), Gd-DTPA (Magnevist), Gd-EOBDTPA (Primovist), Gd-DOTA (Dotarem), and Gd-BTDO3A (Gadovist).* Results*. 32 acute adverse reactions occurred, accounting for 0.3% of all administration. Twelve reactions were associated with Gd-DOTA injection (0.11%), 9 with Gd-BOPTA injection (0.08%), 6 with Gd-BTDO3A (0.056%), 3 with Gd-EOB-DTPA (0.028%), and 2 with Gd-DTPA (0.018%). Twenty-four reactions (75.0%) were mild, four (12.5%) moderate, and four (12.5%) severe. The most severe reactions were seen associated with use of Gd-BOPTA, with 3 severe reactions in 32 total reactions.* Conclusion*. Acute adverse reactions are generally rare with the overall adverse reaction rate of 0.3%. The most common adverse reactions were not severe, consisting in skin rash and hives.

## 1. Introduction

Magnetic resonance imaging (MRI) contrast media (CM) are chelates of gadolinium (Gd), a metal of the lanthanide series with paramagnetic properties. Gd is capable of inducing a strong magnetic field which influences the degree of relaxivity of the protons of water molecules, resulting in a signal increase in MRI [1, 2].

Although the Gd chelates (GBCAs) are safe, adverse reactions induced by their administration have rarely been reported. Adverse events can be classified into two groups: nonallergic reactions (e.g., headache, fatigue, arthralgia, taste perversion, flushed feeling, nausea, or vomiting) and idiosyncratic allergy-like reactions (e.g., hives, diffuse erythema, respiratory distress, chest tightness, respiratory distress, and periorbital edema) [3, 4]. Moreover, depending on the severity, the acute reactions to MRI CM are usually classified as mild, moderate, and severe [3]. Mild reactions are self-limited events, showing no significant progression. These reactions do not need medical treatment, except for the administration of an antihistamine drug to treat skin reactions. On the other hand, moderate reactions require medical treatment in addition to the antihistamine administration or transfer to the emergency clinic. Severe reactions are events that concretely put patient's life to immediate risk. Fortunately, the mild adverse reactions are the most common clinical manifestation, occurring with a rate between 0.07% and 2.4% [3]. Instead, moderate reactions have an incidence of 0.004%–0.7%, and the severe ones, endangering patient's life, rarely exceed a rate of 0.001%–0.01% [3]. These complications are more common in patients with a history of asthma or allergy, in patients who received the contrast agent injected at a faster rate, and in those with history of hypersensitivity to a GBCA or iodinated contrast agents (ICM) [5, 6].

The purpose of our work was to retrospectively evaluate the acute tolerability of the GBCAs in clinical practice of our cancer institute. We reviewed a large number of consecutive patients, considering the severity of their immediate reactions and linking the clinical finding to the specific type of CM employed. All the data were collected according to patient privacy policy.

## 2. Material and Methods

### 2.1. Contrast-Enhanced MRI Studies

From January 2010 to October 2014 there were 10608 Caucasian patients (6.306 men and 4302 women; mean age 61 years; range 21–84 years) undergoing MRI with an intravenous (IV) CM injection performed at our cancer institute. There were 7956 in-patients and 2652 out-patients. MR examinations were performed with a 1.5-T MR system (Magnetom Symphony, upgraded to Total Imaging Matrix Package, Siemens, Erlangen, Germany) using an eighteen-channel body surface phased-array coil. With all studies being performed in the same MR suite, all pre-, intra-, and post-MR steps were made using a constant and homogeneous procedure, as reported in our previous work on the topic [7].

Five different types of MRI CM were employed: Gd-BOPTA (MultiHance), Gd-DTPA (Magnevist), Gd-EOB-DTPA (Primovist), Gd-DOTA (Dotarem), and Gd-BT-DO3A (Gadovist). Chemical structure and properties of MRI contrast agents in our series are showed in Table 1. Choice of GBCAs was made in relation to the type of examination and to the clinical question.  Table 2 indicates the population distribution for each GBCAs.

In 3501 out of 10608 examinations Gd-DOTA was used (in 2806 patients with rectal cancer, in 170 bladder studies, in 95 kidney tumours, in 220 patients with ovarian cancer, in 80 patients with uterus tumour, and in 130 patients with indeterminate mesenteric lesion), in 3002 Gd-BT-DO3A (in 1405 brain studies, in 593 patients with pancreatic lesion, in 650 liver studies, in 220 breast cancers, and in 134 uterus tumours), in 1812 Gd-BOPTA (in 1582 breast tumours and in 230 liver studies), in 1487 Gd-EOB-DTPA for liver and biliary tree studies, and in 806 Gd-DTPA (in 276 patients with sarcomas, in 180 patients with mesenteric lesions, in 270 patients with colorectal cancer, and in 80 patients with neck cancer). The CM were administered IV as a bolus, with a power injector (Spectris Solaris EP MR; MEDRAD, Inc., Indianola, PA) at the standard recommended dosage (0.1 mmol/kg for Gd-EOB-DTPA and Gd-BT-DO3A, 0.2 mmol/kg for Gd-BOPTA, Gd-DOTA, and Gd-DTPA) followed by a 20 mL saline flush. No patient received a double dose or a repeated injection. The injection rate was 2 mL/s.

An informed consent form was accurately filled and signed by each patient, both for MRI examination and for CM injection. For all patients we wrote down personal dates, recent and remote medical history, and any allergies. 1785 patients (16.8%) with a history of allergic asthma or with a previous moderate, or severe, reaction to ICM, or one that included a respiratory component, were submitted to a three-drug prophylaxis scheme with corticosteroids and antihistaminic drugs. This scheme was performed according to the institutional guidelines and adapted from the European guidelines [8]. As the latter, our scheme includes methylprednisolone, one 32 mg tablet orally administered at 12 and 2 hours before the study. In addition, the H1 antihistamines cetirizine, 10 mg orally administered at 36, 12, and 1 hour before the study, and the H2-histamine receptor blocker ranitidine, 50 mg orally administered at 36, 12, and 1 hour before the diagnostic procedure, were used.

Subjects with another kind of hypersensitivity, such as seasonal rhinitis, did not undergo any premedication therapy. Patients were excluded from CM injection in case of history of hypersensitivity reaction to a GBCA and when glomerular filtration rate (GFR) was lower than 30 mL/min/1.73 m^2^. In subjects with a GFR between 30 and 60 mL/min/1.73 m^2^ IV administrating CM was established after a case-by-case analysis. According to our institute procedure, at the end of the MRI examination, all patients were kept under nurse surveillance for the minimum time of two hours and periodically interrogated for any new symptoms and their physical status was checked. All patients who developed adverse reactions were visited by the MRI staff radiologist and, if needed, by the on-call anesthesiologist.

### 2.2. Retrospective Assessment

In our MRI suite all information on contrast-enhanced studies, including the type and volume of the CM administered, the type and severity of any acute adverse reaction, and the need for any pharmacological treatment or resuscitation, is usually collected.

For the purpose of the current study, the adverse reactions were regarded as acute if the symptoms occurred during the first hour from CM administration [9, 10]. As stated by the American College of Radiology manual on CM [3], the severity of adverse reactions to MRI CM was categorized into mild, moderate, and severe reactions; however, we adopted a modified version (Table 3). According to our institute procedure all patients who underwent CM are monitored up to two hours after the end of the examination, so all patients who were subjected to MRI with GBCAs were included in this retrospective study.

### 2.3. Statistical Analysis

Mann-Whitney nonparametric test was used to compare a continuous variable among 2 or more groups. Chi square test with Yates' correction was performed to assess statistically significant difference between percentage values.

A *p* value < 0.05 was considered significant for all tests. All analyses were performed using Statistics Toolbox of Matlab R2007a (The Math-Works Inc., Natick, MA).

## 3. Results

The distribution population of this study can be considered homogenous for several factors: age, gender, patient type, premedication, concomitant drugs, and hypersensitivity (see Table 2). We found 32 (0.3%) reports of acute adverse reactions to all GBCAs among 10,608 contrast-enhanced MRI studies. The reactions occurred in 6 men and 26 women (mean age 53 years; range 32–78 years); in 22 women the CM was administrated for breast study, in 2 for rectal cancer, and in 2 for liver study. Among the men, 4 patients had rectal cancer, one sarcoma, and one pancreatic cancer.

The overall and percontrast medium prevalence and severity of adverse reactions are shown in Table 4. Twenty-four patients developed a mild reaction (75.0%), 4 moderate reactions (12.5%), and 4 severe reactions (12.5%). Seven of these subjects had a seasonal allergic rhinitis history but no one had history of drug or CM allergies/hypersensitivity, so no one was premedicated. No patient had liver or kidney failure.

An adverse reaction was found in 0.34% of the patients receiving Gd-DOTA, 0.5% of the patients receiving Gd-BOPTA, 0.2% of the patients receiving Gd-BT-DO3A, 0.2% of the patients receiving Gd-EOB-DTPA, and 0.25% of the patients receiving Gd-DTPA. No contrast medium had a prevalence of adverse reaction significantly higher or lower than the others. The most common adverse reactions were mild type (24/32), such as skin rash and hives. In addition, we found 4 moderate reactions. Bronchospasm occurred using Gd-BT-DO3A in a patient with pancreatic cancer (man; 51 y), dyspnea with Gd-DOTA in a 38-year-old woman with rectal cancer, symptomatic tachycardia with Gd-DTPA in a 48-year-old man with sarcoma, and mild laryngeal edema with Gd-BOPTA in a 76-year-old woman with breast cancer. We had four of the types being severe. Three of them happened with Gd-BOPTA (one severe respiratory distress, an episode of progressive angioedema, and one of arrhythmia, consisting in supraventricular tachycardia) in women (mean age 63) with breast cancer and another one manifested by using Gd-DOTA (severe respiratory distress) in a 32-year-old man affected by rectal cancer. Nevertheless, no lethal acute reaction was observed.

Although the patients were transferred to the Intensive Care Unit (ICU), all of those were discharged after 24 hours of observation.

The higher percentage of adverse reactions, including the most severe reactions, occurred with Gd-BOPTA, which caused three severe reactions of 32 total cases. However, there was no statistically significant difference between various contrast media used neither was there a prevalence of adverse reaction significantly higher or lower related to patients age or the use of drugs as aspirin or chemotherapeutic agents.

## 4. Discussion

In our study the rate of acute adverse reaction to GBCAs was 0.3%, in a time period of about five years. This is in agreement with the data from the published literature [3, 4]. As a matter of fact, GBCAs are considered safe, with a rate of acute adverse reactions less than 1% in several retrospective analyses [11]. Most of the acute adverse reactions occurring in our study were mild reactions, represented mainly by skin reactions, such as hives or rash, and this observation represents a confirmation of the data from other series [11, 12].

An interesting topic regards the rate of adverse events between various CM used. In our analysis the frequency of adverse reactions was slightly higher using Gd-BOPTA (0.5%) than with the other four agents (0.2%–0.3%), although this difference was not significant. The most severe reactions were more frequent using Gd-BOPTA, but the difference with the other CM was not statistically significant. In agreement with our study, Kirchin and Runge reported a similar overall incidence and type of adverse events using different contrast agents [13]. Also, Prince et al. reported that Gd-BOPTA had a significantly higher rate of adverse events and the highest rate of arrests compared with Gd-DTPA-BMA and Gd-DTPA, nonionic linear GBCAs [14].

According to our findings, age did not affect the frequency and severity of reactions with any specific CM, which is in agreement with Jung et al. [15]. On the contrary, they were some reports suggesting that severity may be higher in young patients [12]. About the gender we found that adverse reactions to MRI contrast agents were more frequent in women (81.25%) than in man (18.75%), which is also in agreement with previous reports [15, 16]. Conversely to Jung et al. [15], and Aran et al. [16], we showed that the severe reactions were more frequent in women (75%) than in man (15%); however, the data is not statistically significant. The data by Jung et al. [15] suggest that men are more likely to die as a result of acute adverse events; in our series no patients died. Although 4 patients were transferred to the ICU, all of those were discharged after 24 hours of observation.

The safety of GBCAs is mostly based on their stability in vivo. The main source of concern about the stability of GBCAs is represented by transmetallation. The reaction of transmetallation consists in the substitution of ions by chelating copper and zinc in the body, leading to the release of free Gd ions [13, 17–20]. The stability of Gd chelates depends on their chemical structure. The molecular structure, whether cyclic or linear, and the ionicity determine the stability of Gd chelates. Linear chelates are flexible open chains which do not offer a strong binding to Gd3+. In contrast, the macrocyclic chelates offer a strong binding to Gd3+ by the virtue of being preorganized rigid rings of almost optimal size to cage the Gd atom. Nonionic preparations are also less stable in comparison to the ionic ones as the binding between Gd3+ with the negatively charged carboxyl groups is stronger in comparison to that with amides or alcohol in the nonionic preparations. According to stability constants and kinetic measurements, the most stable GBCA is the ionic-macrocyclic chelate Gd-DOTA and the least stable agents are the nonionic linear chelates gadodiamide and gadoversetamide [17–20]. In vivo data confirmed the low stability of linear nonionic chelates but no significant difference was observed among the macrocyclic molecules, both ionic (Gd-DOTA) and nonionic (Gd-HP-DO3A and Gd-BTDO3A) [14, 19, 20]. In our large cohort of study no subjects received linear nonionic CM. Considering the macrocyclic molecules, although the most severe reactions were more frequent using Gd-BOPTA, the difference with the other CM was not statistically significant. These results are in accordance with those compiled in previous reports [17–20]. Thermodynamic/kinetic stability data do not play any role regarding the risk of hypersensitivity, whereas stability of GBCAs is more involved in the development of nephrogenic systemic fibrosis (NSF) and in the Gd deposition in neural structures, such as the dentate nucleus and globus pallidus. It may be, on the other hand, hypothesized that the propensity of Gd-BOPTA to bind proteins may favour the risk of triggering hypersensitivity reactions [21–23].

In our series two patients had cardiovascular modifications: one a moderate reaction (symptomatic tachycardia with Gd-DTPA) and the other a severe reaction (supraventricular tachycardia with Gd-BOPTA). Several studies on animal models demonstrated that GBCAs IV injected can cause cardiovascular modifications, including transient blood pressure disturbances, nonspecific electrocardiogram (ECG) changes, prolonged PR interval, tachycardia, atrioventricular conduction defects, and atrial and ventricular arrhythmias [24–26]. As showed by Idée and colleagues, the transient Gd-HP-DO3A induced rise in blood pressure is probably the consequence of a positive inotropic effect [24]. Moreover, cardiodepressive and hypotensive effects caused by gadopentetate dimeglumine are dose-dependent [24, 26]. This data was confirmed by Pirovano et al. who showed that the injection of 0.2 mmol/kg gadobenate dimeglumine had no detrimental effect on cardiac electrophysiology, or other safety parameters, in healthy volunteers or patients with coronary artery disease [27].

Of the 32 adverse reactions in our series, 21% occurred in patients with a history of seasonal allergic rhinitis, in the absence of any premedication drug, while in no one premedicated patient did we find any reaction. Furthermore, Nelson et al. reported that the risk of adverse reaction to GBCAs is approximately eight times higher in patients who had previous adverse reactions to Gd and the severity of the second episode is generally higher than the first one [5]. In our study no patients showing severe adverse reactions had a story of previous adverse reactions to Gd or previous hypersensitivity reactions to other CM. However, the frequency of reactions was greater in those with seasonal allergic rhinitis history. Similarly, Aran et al. showed that patients with underlying allergic diseases and previous reactions had a frequency and severity greater than others [16]. In our analysis, we found that premedicated patients, such as those with history of previous reactions to drugs, did not show adverse reactions. These findings suggest the role of immunological mechanisms that lead to adverse reactions in those with underlying allergic disease, according to other studies [5, 16]. So, assuming that the frequency of adverse reactions to GBCAs is about 2.3 to 3.7 times higher in patients with a history of reactions to ICM, GBCAs should be used with caution in this setting of patients, especially in case of previous severe reactions [28]. The role of premedication with corticosteroids as a preventive measure in patients with a history of reaction to GBCAs is not well established [5, 29]. In this group of patients, although there is no scientific evidence of the effectiveness of premedication, Murphy et al. recommended both a premedication with corticosteroids during the 24 hours before doing an MRI examination with CM and the use of a different CM, as preventive measures [29]. According to our institutional policy, we use a prophylactic drug scheme. Moreover, if a patient shows hypersensitivity to GBCAs the diagnostic procedure will be performed using a different CM or without CM, when possible.

Several limitations are recognizable in the design of our study. The project was a retrospective review of the incidence, so some minor reactions may not have been identified and reported.

## 5. Conclusion

GBCAs are safe and well tolerated by patients; in our study rate of acute adverse reactions is generally rare with the overall adverse reaction rate of 0.3%, and we should evaluate the data in relation to the use of accurate premedication in case of history of previous reactions to drugs or ICM. The most frequent reactions (24 out of 32) were mild type, without lethal events. The higher percentage of adverse reactions (0.5% versus 0.2–0.3%) and the most severe reactions occurred with Gd-BOPTA; however there is no statistically significant difference between various MRI CM used.

## Figures and Tables

**Table 1:** Chemical structure and properties of MRI contrast agents in our series.

Trademark	Contrast medium	Molecular structure
MultiHance 0.5 mol/L (Bracco, Milan, Italy)	Gd-BOPTA (gadobenate dimeglumine)	Linear, ionic
Primovist 0.25 mol/L (Bayer Schering Pharma AG, Berlin, Germany)	Gd-EOB-DTPA (gadoxetic acid disodium)	Linear, ionic
Dotarem 0.5 mol/L (Guerbet Research, Aulnay-sous-Bois, France)	Gd-DOTA (gadoterate meglumine)	Macrocyclic, ionic
Gadovist 0.5 mol/L (Bayer Schering Pharma AG, Berlin, Germany)	Gd-BT-DO3A (gadobutrol)	Macrocyclic, nonionic
Magnevist 0.5 mol/L (Bayer Schering Pharma AG, Berlin, Germany)	Gd-DTPA (gadopentetate dimeglumine)	Linear, ionic

Note: Gadovist, Primovist, and Magnevist are from Bayer Healthcare, Berlin, Germany; Dotarem is from Guerbet, Villepinte, France.

**Table 2:** Population distribution for each GBCA.

	Gd-BT-DO3A	Gd-DOTA	Gd-BOPTA	Gd-DTPA	Gd-EOB-DTPA	*p* value
Age (mean ± range)	59 (22–79)	62 (29–78)	56 (22–82)	60 (22–84)	61 (29–86)	0.21
Gender (number of men/women)	1771/1231	2106/1395	1051/761	483/323	895/592	0.59
No patients	3002	3501	1812	806	1487	
Patient type (number of inpatients/outpatients)	2350/652	2766/735	1409/403	624/182	1159/328	0.76
No patients premedicated	510	596	299	136	244	0.89
No patients using concomitant drugs	1350	1610	779	378	661	0.24
Iodinated CM hypersensitivity	158	201	74	40	70	0.14
MRI examination type (abdomen/brain/head & neck/breast)	1377/1405/0/220	3501/0/0/0	230/0/0/1582	726/0/80/0	1487/0/0/0	<0.001

**Table 3:** Classification of severity and manifestations of acute reactions to MR contrast media (modified from [3]).

Type of reaction	Features
Mild	Rash, itch, cough, hives, sneezing, nasal stuffiness, mild eye swelling, mild facial swelling, vomiting, nausea, perspiration, warmth, anxiety, flushing, altered taste
Moderate	Dyspnea, bronchospasm, symptomatic tachycardia, symptomatic bradycardia, mild laryngeal edema, hypotension
Severe	Severe respiratory distress, responsiveness, arrhythmia, convulsion, cardiopulmonary arrest, progressive angioedema, marked hypotension

Notes: mild = nonmedical intervention with exception of possible antihistamine administration; moderate = requiring immediate medical treatment or transfer to emergency department; severe = life-threatening, potentially fatal event typically requiring hospitalization if an outpatient.

**Table 4:** Overall and percontrast medium prevalence and severity of adverse reactions.

	Gd-BT-DO3A	Gd-DOTA	Gd-BOPTA	Gd-DTPA	Gd-EOB-DTPA	*Total*	*p value* ^*∗*^
	Number (%)	Number (%)	Number (%)	Number (%)	Number (%)		
Mild reactions	5 (0.17)	10 (0.29)	5 (0.28)	1 (0.12)	3 (0.20)	24 (0.75)	0.053
Moderate reactions	1 (0.03)	1 (0.029)	1 (0.055)	1 (0.12)	0 (0)	4 (0.125)	0.91
Severe reactions	0 (0)	1 (0.029)	3 (0.17)	0 (0)	0 (0)	4 (0.125)	0.07

*Total*	6 (0.2)	12 (0.34)	9 (0.5)	2 (0.25)	3 (0.20)	32 (0.3)	

*p value*	0.03	0.001	0.26	0.61	0.05		0.038

**^*∗*^ *χ* ^2^.
